# Post traumatic stress disorder among school children of Army Public School Peshawar after Six month of terrorists attack

**DOI:** 10.12669/pjms.343.14885

**Published:** 2018

**Authors:** Aftab Khan, Obaid Ullah, Khadija Nawaz, Arsalan, Ambreen, Israr Ahmad

**Affiliations:** 1 Aftab Khan, M.Phil. Pakistan Medical Research Council (PMRC) Research Centre, Khyber Medical College, Peshawar, Pakistan; 2 Obaid Ullah M.Phil. Pakistan Medical Research Council (PMRC) Research Centre, Khyber Medical College, Peshawar, Pakistan; 3 Khadija Nawaz, MSC Psychology. Child protection and welfare commission, overnment of Khyber Pakhtunkhwa, Pakistan; 4 Arsalan Inayat, MBBS. Khyber Teaching Hospital, Peshawar, Pakistan; 5 Ambreen, M.Phil. Pakistan Medical Research Council (PMRC) Research Centre, Khyber Medical College, Peshawar, Pakistan; 6 Israr Ahmad, Ph.D. Pakistan Medical Research Council (PMRC) Research Centre, Khyber Medical College, Peshawar, Pakistan

**Keywords:** PTSD, CPSS, Terrorist, Functional impairment, Therapies, Trauma

## Abstract

**Background & Objectives:**

Terrorist attack in Army Public School Peshawar, Pakistan left behind more than hundred children dead. It was the highest death toll of children in the world in a single terrorists attack. The attack dominated national and international news, high level security measures have been adopted in all school throughout Pakistan, which created fear and stress in children. The objective of the study was to determine post-traumatic stress disorder among children after six month of terrorist attack inspite of rigorous psychosocial support and rehabilitation.

**Methods:**

We wanted to determine Post Traumatic Stress Disorder (PTSD) among children of Army Public School of age range 10 to 18 years after 5 months of intervention and rehabilitation following terrorists attack. For this a self-report questionnaire, Child PTSD Symptom Scale (CPSS), which assess and identify symptoms matching DSM (Diagnostic and Statistical Manual of Mental Disorders) IV criteria for post-traumatic stress disorder of children, was filled. Informed consent was taken from school Principal and responders.

**Results:**

A total 205 students of age range 10 to 18 years participated in the study. The most frequent age group of the study were 16 years and 14 years students with frequency 58 (28.3%) and 46 (22.4%) respectively. Among 205 participated school children PTSD were found in 154 (75.2%) children while only 24.8% students had no PTSD symptoms. In more than 50% PTSD positive school children had functional impairment for each category of fun and hobbies, friendship, school work, family relation, doing chores, general happiness and saying prayers.

**Conclusion:**

Study found a very high prevalence of PTSD among 10 to 18 years age group students of Army Public School inspite of five months continuous intervention and rehabilitation services. Study showed that this age group needs long term psychosocial treatment in case of trauma.

## INTRODUCTION

Terrorism and war are the acts of violence emerged with the perpetrated act of human. The impact of war and terrorism occur as the direct result of physical, media exposure and visual impact or through the various form of interpersonal experiences. Intergroup conflict are ancient in the history of world, it estimated that two million children have been killed due to war related injuries, one million orphaned, four million have been disabled and more than 12 million has been displaced from their homes.^1^

Number of natural and manmade disaster put very devastating psychological impact on the eye witnesses, who may be children. Think of a child who has witnessed the loss of his family members in a war or a natural disaster, and his flash back memories of such event could be very traumatic for that child and need urgent medical attention. Post traumatic stress disorder is a psychiatric disorder that a person may develop after witnessing or experiencing another person receiving actual or potential harm including serious bodily injury or sexual abuse.^2^

World is experiencing global spread of terrorism these days. Pakistan is the worse terrorism affected country of the world. Major cities of Pakistan were effected due to terrorism but Peshawar city witnessed very high death toll due to terrorism.^3^ Pakistanis are at highest risk group for developing traumatic stress reactions. Throughout its history, Pakistani citizen have faced many losses in term of lives and material in the form of terrorism in many types of violence, such as political, secessionist, sectarian, ethnic and recently religious fundamentalist. Terrorist attacks staged in Pakistan have killed over 35000 people caused material damage to the Pakistani economy totaling greater than $67 billion.^4^

In the streamline of terrorism in Pakistan Terrorists attack in Army public school Peshawar on 16 December 2014, left behind more than hundred children dead. It was the highest death toll of children in the world in a single terrorists attack. The attack dominated national and international news, high level security measures have been adopted in all school throughout Pakistan, which created fear and stress in children. Majority of children starts experiencing mental stress, depression, anxiety and shock. Such kind of incident left unforgettable and most horrifying impact in their minds which results in psychological disorder known as Post Traumatic Stress Disoder.^5^ Trauma impacts children differently at each developmental stage. In order to understand the constellation and severity of PTSD symptoms, it is important to acknowledge the age at which a person experiences a traumatic event.^6^ Children exposed to traumatic experiences at early ages are at risk for a variety of psychiatric problems.^7^ Children may be diagnosed with specific psychiatric illnesses (e.g. ADHD, ODD separation anxiety, etc.), but the true underlying issue may be PTSD.

What happened in Army Public School (APS) Peshawar on December 16, 2014 is no less than a traumatic event witnessed in a war setting, say psychologists and psychiatrists. Schoolchildren across the country were experiencing fear as threats continue to emerge in different cities. Parents are reporting children who survived the APS massacre are displaying low-levels of interest in going back to school and in other activities.^8^

After the tragic and horrifying event of Army Public School number of government and non-government organizations approached and started their interventions through counseling, therapies, medical care’s and relaxation techniques. A similar research looked at the psychometric responses of 358 adolescents randomly selected from war effected Gaza Strip. It concluded that apart from 11.8% of the sample, the rest displayed symptoms or full onset of PTSD.^9^ Our objective was to determine post-traumatic stress disorder among children after six month of terrorist attack in spite of rigorous psychosocial support and rehabilitation.

## METHODS

This cross sectional descriptive study was carried out among all surviving school children of Army Public School Peshawar, Pakistan of age range 10 to 18 year during the month of May & June 2015. A self-report questionnaire, Child PTSD Symptom Scale (CPSS),^10^ which assess and identify symptoms matching DSM (Diagnostic and Statistical Manual of Mental Disorders) IV criteria for post-traumatic stress disorder of children, was filled after taking informed consent from participants. Ethical approval had been granted by the Ethical Review Board Post Graduate Medical Institute Hayatabad.

The trained interviewer including the panel of psychologist and researchers visited army public school and invited the students falling in the inclusion criteria to participate in the study. Psychologist took session of each students applying relaxation technique for sensible response to the questionnaire. The purpose and importance of the study was described to each participant.

CPSS scale determine the severity of PTSD symptoms consist of 24 questions, each of the first 17 items is rated on a scale from 0 to 3, with total score ranging from 0 to 51 by adding them up. Items 1-5 are re-experiencing symptoms, items 6-12 are avoidance symptoms, and items 13-17 are hyper-arousal symptoms.

A guide to PTSD severity based on the total scores was determined as 0 – 10 (Below threshold) 11 – 15 (Subclinical – Mild) 16 – 20 (Mild) 21 – 25 (Moderate) 26 – 30 (Moderately Severe) 31 – 40 (Severe) 41 – 51(Extremely Severe).

The additional seven items that inquire about daily functioning (e.g., relationships with friends, schoolwork) are rated as either absent (0) or present (1) and yield a total impairment severity score ranging from 0 to 7.

### Statistical Analysis

Data was analyzed using SPSS version 16.0. Frequencies of demographic variables etc. were calculated. Frequency of PTSD was calculated by using a cut off value of ≥ 14.

## RESULTS

**Fig.1:**
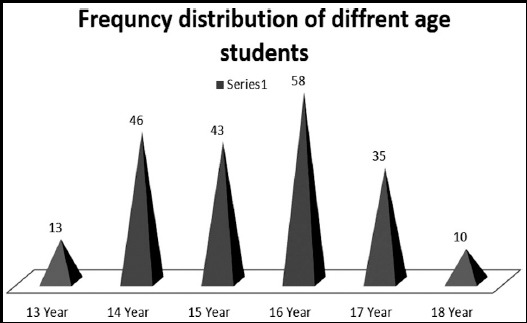
Age wise distribution of participated students.

A total of 205 school children of Army public school (APS) of age range 10 to 18 years were included in the study. Among 205 students 5% students were of class 8^th^ and 31%, 37%, 17% and 10% students were of class 9^th^, 10^th^, 11^th^ and 12^th^ respectively. The most frequent age group of the study were 16 years and 14 years students with frequency 58 (28.3%) and 46 (22.4%) respectively (Fig.1). Among 205 who participated school children PTSD were found in 154 (75.2%) children while only 24.8% students had no PTSD symptoms (Table-I). It was found that in 20.5% children PTSD score were more than 40 and was very seriously affected with the trauma. In more than 50% PTSD positive school children had functional impairment for each category of fun and hobbies, friendship, school work, family relation, doing chores, general happiness and saying prayers. General happiness was equally affected both in PTSD and non PTSD children (Table-II).

**Table-I:** Frequency of PTSD among school children of Army Public School Peshawar.

S#	Finding	Frequency
1	No PTSD	51 (24.8%)
2	PTSD Positive	154 (75.2%)
3	Severe PTSD	42 (20.5%)

**Table-II:** Frequency of functional impairment among APS students after terrorist attack.

S#	Functions	Non PTSD students N=51	In PTSD students N=154
1	Fun and Hobbies	24 (47%)	103 (67%)
2	Friendship	22 (43%)	98 (63.6%)
3	School work	24(47%)	88 (57%)
4	Family Relationship	20 (39%)	77 (50%)
5	Doing Chores	17 (33%)	76 (49.3%)
6	General Happiness	31 (60.7%)	98 (63.6%)
7	Saying prayers	21 (41%)	91 (59%)

## DISCUSSION

This research survey demonstrate the impact of rehabilitation and psychotherapy services carried out in Army public school for children after Six month of terrorists attack. Although other psychological difficulties may be present but this study focused only on the diagnosis of PTSD in children based on diagnostic criteria of DSM IV using CPSS scale for children.

The principal finding of our study was that 75.2% children of age range 10-18 years of APS School were suffering from PTSD despite five to six month of governmental and nongovernmental organizations psychotherapy, counseling, relaxation techniques and rehabilitation services. However another study shows that 23.6% of the people of Khyber Pakhtunkhwa (KPK) suffer from PTSD as a result of terrorism.^4^ In July 2011 Oslo bombing on the government ministries of Norway left behind many casualties and more than 100 injured. After 10 month of this attacked a PTSD survey among government employees showed 24% PTSD prevalence^11^ which was very low than APS students comparatively. Major determinants of high rate of PTSD among APS school children was age factor, global media coverage of the event and high security measures which flash back memories continuously.

The other important factors of PTSD than physical exposure are relationship to direct victims, bomb-related television viewing, and lingering safety concerns and worry in predicting PTSD reactions. These findings are consistent with other studies^12^,^13^ and support the inclusion of the child’s subjective experience at the time of exposure in the diagnostic criteria for PTSD.

The study included children of age range 10 to 18 years, mostly participants were of class 8 to class 12. The victim of the event was this age group children and these block of children was the eye witness of the event while junior class was settled in other block which was evacuated at the time of attack. Main focus of this study was on the directly affected children who lost their friends.

It was found that more than 50% PTSD children had functional impairment such as fun and hobbies, friendship, school work, family relation, doing chores, general happiness and saying prayers. The relationship between PTSD and work has not been investigated to the same extent as for depression, particularly in relation to reduced productivity and performance on the job. However few studies have suggested that PTSD is associated with impaired occupational functioning, as indicated by unemployment, absenteeism, work disability and lack of fun & hobbies.^14^-^16^ Another study explored specific pathways to impairment, found that that PTSD re-experiencing and hyperarousal symptoms were significantly correlated with work impairment.^17^,^18^

In addition, some of the other PTSD symptoms were not associated with work impairment, even though they were correlated with social and relationship functioning. This study showed almost equal rate of functional impairment (Table-II) in both PTSD and non PTSD children which justified the fact that PTSD symptoms have a little impact on functional impairment. Slight differences in functional impairment have been observed in this study between PTSD and non PTSD children.

### Limitations of the study

First our sample was not a randomized because very few children survived. Second our sample was not differentiated as exposed and non-exposed due to limited time access in the APS School. Third we used single item scales for assessing the PTSD level. Owing to logistic constrains of assessing other aspect Psychological abnormalities during such stressful period we used brief instrument. Single item scales of risk factors have been used in previous studies and were shown to predict PTSD.^19^

Despite these limitations this study may have fruitful finding in such a frightful, security zone and competitive atmosphere. The study provided useful recommendation that this age group need long term psychological treatment and rehabilitation services in case of trauma.
